# Comparative carcinogenicity study of a thick, straight-type and a thin, tangled-type multi-walled carbon nanotube administered by intra-tracheal instillation in the rat

**DOI:** 10.1186/s12989-020-00382-y

**Published:** 2020-10-15

**Authors:** Dina Mourad Saleh, William T. Alexander, Takamasa Numano, Omnia Hosny Mohamed Ahmed, Sivagami Gunasekaran, David B. Alexander, Mohamed Abdelgied, Ahmed M. El-Gazzar, Hiroshi Takase, Jiegou Xu, Aya Naiki-Ito, Satoru Takahashi, Akihiko Hirose, Makoto Ohnishi, Jun Kanno, Hiroyuki Tsuda

**Affiliations:** 1 Nanotoxicology Project, Nagoya City University, 3-1 Tanabe-Dohri, Mizuho-ku, Nagoya, 466-8603 Japan; 2 Department of Experimental Pathology and Tumor Biology, Nagoya City University Graduate School of Medical Sciences, Nagoya, Japan; 3 Department of Forensic Medicine and Clinical Toxicology, Faculty of Medicine, Assuit University, Assuit, Egypt; 4 Department of Forensic Medicine and Clinical Toxicology, Faculty of Medicine, Aswan University, Aswan, Egypt; 5 Department of Forensic Medicine and Toxicology, Faculty of Veterinary Medicine, Beni-Suef University, Beni-Suef, Egypt; 6 Department of Veterinary Toxicology, Faculty of Veterinary Medicine, Alexandria University, Alexandria, Egypt; 7 Core Laboratory, Nagoya City University Graduate School of Medical Sciences, Nagoya, Japan; 8 Department of Immunology, Anhui Medical University College of Basic Medical Sciences, Hefei, China; 9 Division of Risk Assessment, National Institute of Health Sciences, Kawasaki, Japan; 10 Japan Industrial Safety and Health Association, Japan Bioassay Research Center, Hadano, Kanagawa Japan

**Keywords:** MWCNT, Carcinogenicity, Thick and thin, Intratracheal, Intrapulmonary

## Abstract

**Background:**

Multi-walled carbon nanotubes can be divided into two general subtypes: tangled and straight. MWCNT-N (60 nm in diameter) and MWCNT-7 (80–90 nm in diameter) are straight-type MWCNTs, and similarly to asbestos, both are carcinogenic to the lung and pleura when administered to rats via the airway. Injection of straight-type MWCNTs into the peritoneal cavity also induces the development of mesothelioma, however, injection of tangled-type MWCNTs into the peritoneal cavity does not induce carcinogenesis. To investigate these effects in the lung we conducted a 2-year comparative study of the potential carcinogenicities of a straight-type MWCNT, MWCNT-A (approximately 150 nm in diameter), and a tangled-type MWCNT, MWCNT-B (7.4 nm in diameter) after administration into the rat lung. Crocidolite asbestos was used as the reference material, and rats administered vehicle were used as the controls. Test materials were administered by intra-Tracheal Intra-Pulmonary Spraying (TIPS) once a week over a 7 week period (8 administrations from day 1 to day 50), followed by a 2-year observation period without further treatment. Rats were administered total doses of 0.5 or 1.0 mg MWCNT-A and MWCNT-B or 1.0 mg asbestos.

**Results:**

There was no difference in survival between any of the groups. The rats administered MWCNT-A or asbestos did not have a significant increase in bronchiolo-alveolar hyperplasia or tumors in the lung. However, the rats administered MWCNT-B did have significantly elevated incidences of bronchiolo-alveolar hyperplasia and tumors in the lung: the incidence of bronchiolo-alveolar hyperplasia was 0/20, 6/20, and 9/20 in the vehicle, 0.5 mg MWCNT-B, and 1.0 mg MWCNT-B groups, respectively, and the incidence of adenoma and adenocarcinoma combined was 1/19, 5/20, and 7/20 in the vehicle, 0.5 mg MWCNT-B, and 1.0 mg MWCNT-B groups, respectively. Malignant pleural mesothelioma was not induced in any of the groups.

**Conclusions:**

The results of this initial study indicate that tangled-type MWCNT-B is carcinogenic to the rat lung when administered via the airway, and that straight-type MWCNT-A did not have higher carcinogenic potential in the rat lung than tangled-type MWCNT-B.

## Background

Multi-walled carbon nanotubes (MWCNTs) are composed of multiple coaxially arranged graphene cylinders. The sp2 bonded carbon atoms of the graphene cylinders give MWCNTs remarkable mechanical, chemical, physical, and electrical properties, making these materials highly useful in a variety of applications [1–4]. MWCNTs range in diameter from 2 to over 100 nm depending on the number of graphene cylinders i.e., the number of walls, that compose the MWCNT [1, 5]. MWCNTs can be divided into two general subtypes: tangled and straight. MWCNTs with low wall numbers are flexible and can assemble into tangled agglomerates. As wall number increases, the MWCNT becomes more rigid and straight.

Fibrous materials such as asbestos are known to be harmful to the respiratory tract causing persistent inflammatory lesions and eventually inducing neoplastic development [6, 7]. Initially, the straight-type MWCNT, MWCNT-7 (also known as Mitsui MWCNT-7 and MWNT-7 [8–10]), with 40 walls and a diameter of approximately 100 nm, was shown to induce mesothelioma after intraperitoneal or intrascrotal administration in rats and mice [9, 10]. Based in part on these findings, WHO/International Agency for Research on Cancer (IARC) evaluated MWCNT-7 as “Sufficient Evidence of Carcinogenicity in Experimental Animals” and thus “Possibly Carcinogenic to Humans (Group 2B)” [6].

Studies using intraperitoneal and intrascrotal administration cannot be extrapolated directly to human risk; therefore, to study the toxic effects of MWCNTs in the lung and pleura after administration via the airway, we conducted short term studies with MWCNT-7 and another straight-type MWCNT, MWCNT-N (Nikkiso: 30 layers, approximately 60 nm in diameter). Crocidolite asbestos (UICC Grade) was used as a reference material [11]. Test materials were administered using intra-Tracheal Intra-Pulmonary Spraying (TIPS). We found hyperplastic proliferative lesions of the visceral mesothelium in rats administered MWCNT-N, MWCNT-7, and crocidolite. Based on these results, we conducted a 2-year study with MWCNT-N: MWCNT-N was administered using TIPS followed by a 2-year observation period. We found that MWCNT-N induced development of bronchiolo-alveolar and pleural tumors in rats [12]. In two other studies, rats exposed to MWCNT-7 (lot No. 071223 fibers had an average diameter of 83.8 nm and lot No. 080126 fibers had an average diameter of 90.7 nm) for 2 years by whole body inhalation developed lung tumors [8], and rats administered MWCNT-7 by TIPS developed pleural mesotheliomas [13]. These results indicate that rigid straight-type MWCNTs can be carcinogenic to the lung and pleural mesothelium in the rat after administration to the lung.

Studies of MWCNTs administered by intraperitoneal injection or intraperitoneal implantation report a general difference in the carcinogenicity of tangled and straight-type MWCNTs. Thin tangled-type MWCNTs did not induce mesotheliomas when administered into the peritoneal cavity [14–17]; an intermediate-type MWCNT had relatively low carcinogenic potential when administered into the peritoneal cavity [18]; and rigid, straight-type MWCNTs generally had high carcinogenic potential when administered into the peritoneal cavity [16–19].

The results of the studies cited above suggest that thin tangled-type MWCNTs may be less carcinogenic than straight-type MWCNTs. However, there are no reports of the carcinogenicity of thin tangled-type MWCNTs in the lung. Therefore, we conducted a preliminary subchronic study to study the effects of a thick straight-type MWCNT, MWCNT-L, and a thin tangled-type MWCNT, MWCNT-S, in the lung and pleura after administration via the airway. Rats were administered MWCNTs at a dose of 0.125 mg/rat once every 2 weeks over a 24 week period by TIPS. We found that the straight-type MWCNT caused mesothelial proliferative lesions while the tangled-type induced marked inflammation in lung tissue [20]. These results suggested that the straight-type MWCNT was possibly more active in the mesothelium and the tangled-type MWCNT was possibly more active in the lung. Therefore, we conducted the present long-term study using TIPS administration of MWCNT-A, a thick straight-type MWCNT approximately 150 nm in diameter and composed of 213 walls, and MWCNT-B, a thin tangled-type MWCNT approximately 7.4 nm in diameter and composed of 6–7 walls, to investigate the relevance of the rigidity and shape of MWCNT fibers administered via the airway to MWCNT-induced lung and pleural carcinogenesis.

## Results

### MWCNTs used in this study

The lengths and diameters of airborne fibers prior to homogenization and in vehicle after homogenization are shown in Table 5 and Additional file 1. Homogenization of MWCNT-A in the vehicle had little effect on its length or diameter measurements. Homogenization of MWCNT-B dramatically decreased both its length and diameter measurements, indicating disruption of larger agglomerates.

### Interim sacrifice at week 52

Five rats from each group were sacrificed at week 52. No macroscopic lesions were found. In the rats administered MWCNT-A and MWCNT-B there was a dark gray discoloration of the lung and parabronchial and mediastinal lymph nodes due the presence of MWCNTs. Microscopically, rats administered MWCNT-A and MWCNT-B had chronic inflammatory lesions throughout the lung with some fibrous thickening of the alveolar wall. Macrophages engulfing MWNCT fibers were also present in the lungs of these rats. Induction of bronchiolo-alveolar hyperplasia was observed in 3 rats in the 0.5 mg MWCNT-A group and in all 5 rats in the 1.0 mg MWCNT-A group, the 0.5 mg and 1.0 mg MWCNT-B groups, and the 1.0 mg crocidolite group (Table 1). No tumors were found in the lung, mesothelium, or other organs.

**Table 1:** Incidence of bronchiolo-alveolar cell hyperplasia in rats sacrificed at week 52

Treatment Group	Number of rats examined	Number of rats with bronchiolo-alveolar hyperplasia
Vehicle	5	0
MWCNT-A (0.5 mg)	5	3
MWCNT-A (1.0 mg)	5	5**
MWCNT-B (0.5 mg)	5	5**
MWCNT-B (1.0 mg)	5	5**
Crocidolite	5	5**

** *p* < 0.01

### Terminal sacrifice at week 104

No significant difference was observed in overall survival by Kaplan-Meyer analysis (not shown). The mean survival time for the groups is shown in Table 2. One animal in the vehicle group became moribund and was sacrificed at 39 weeks due to bleeding in the brain associated with a pituitary tumor; therefore the effective number of rats in this group was 19. The number of rats in the other groups was 20.

**Table 2:** Incidence of bronchiolo-alveolar cell hyperplasia and lung tumors in rats sacrificed at week 104

	Vehicle	MWCNT-A		MWCNT-B		Crocidolite
		0.5 mg	1.0 mg	0.5 mg	1.0 mg	1.0 mg
Animals examined	19	20	20	20	20	20
Mean Survival (wks)	98.2	102.2	100.4	95.6	100.0	97.9
Hyperplasia	0	3	2	6*	9**	4
Adenoma	1	4	1	4	5	3
Carcinoma	0	1	3	1	2	0
Adenoma, Carcinoma Combined	1	5	4	5	7*	3

*,** *p* < 0.05, *p*<0.01

### MWCNTs in the lung tissue

**Fig. 1:**
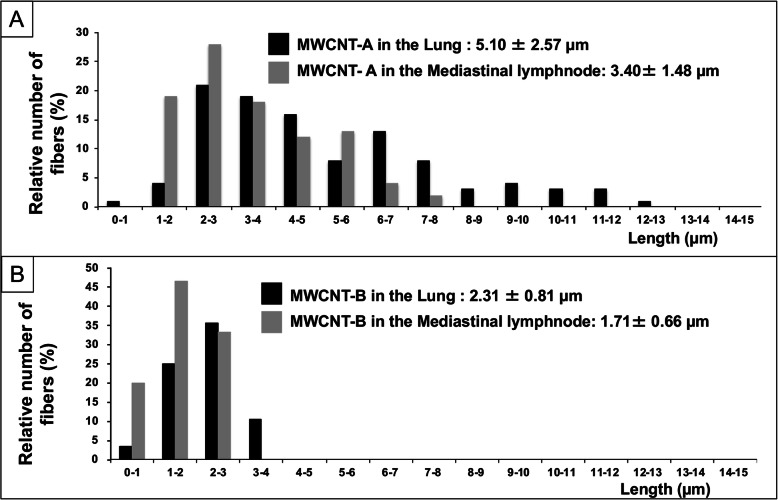
**a** Length distribution of MWCNT-A in the lung alveoli and mediastinal lymph nodes. **b** Length distribution of MWCNT-B in the lung alveoli and mediastinal lymph nodes

The length distributions of MWCNT-A and MWCNT-B in the lung and mediastinal lymph node is shown in Fig. 1. The mean length of MWCNT-A was 5.10 ± 2.57 μm in the lung and 3.40 ± 1.48 μm in the mediastinal lymph node. The mean length of MWCNT-B was 2.31 ± 0.81 μm in the lung and 1.71 ± 0.66 μm in the mediastinal lymph node.

**Fig. 2:**
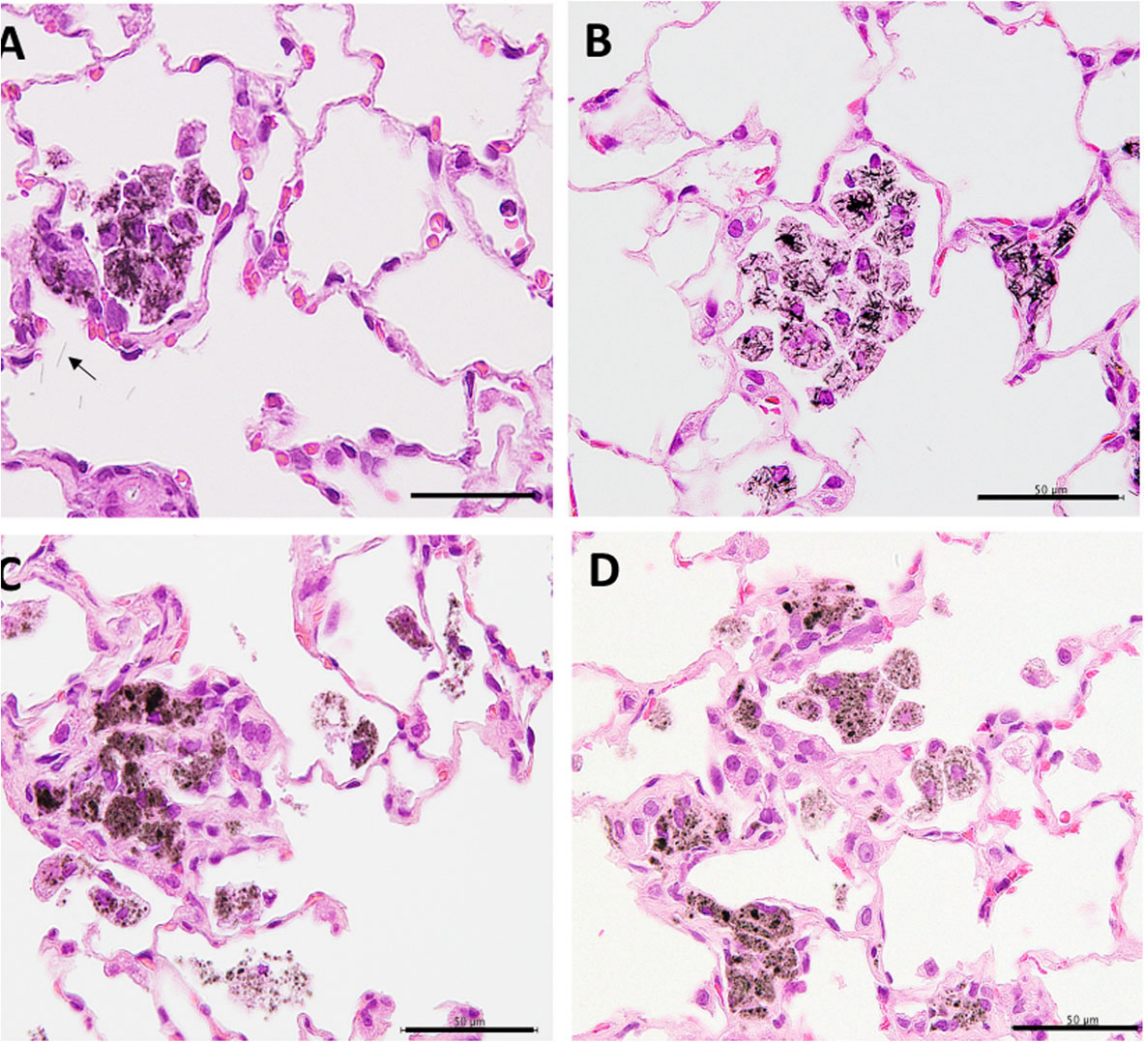
H&E sections of the lungs of rats treated with MWCNT-A and MWCNT-B. **a** MWCNT-A in a rat lung at 52 weeks. MWCNT-A is straight-shaped. Fibers in alveolar macrophages can be seen. Arrow points to a free fiber. **b** MWCNT-A in a rat lung at 104 weeks. Fibers in macrophages and in granulation tissue can be seen. **c** MWCNT-B in a rat lung at 52 weeks. MWCNT-B has a coarse granular appearance in the cytoplasm of alveolar macrophages. Fibers in granulation tissue and in free macrophages can be seen. **d** MWCNT-B in a rat lung at 104 weeks. Fibers are mostly encased in granulation tissue. Macrophage aggregates and free macrophages with MWCNT-B fibers can also be seen. Scale bar represents 50 μm

**Fig. 3:**
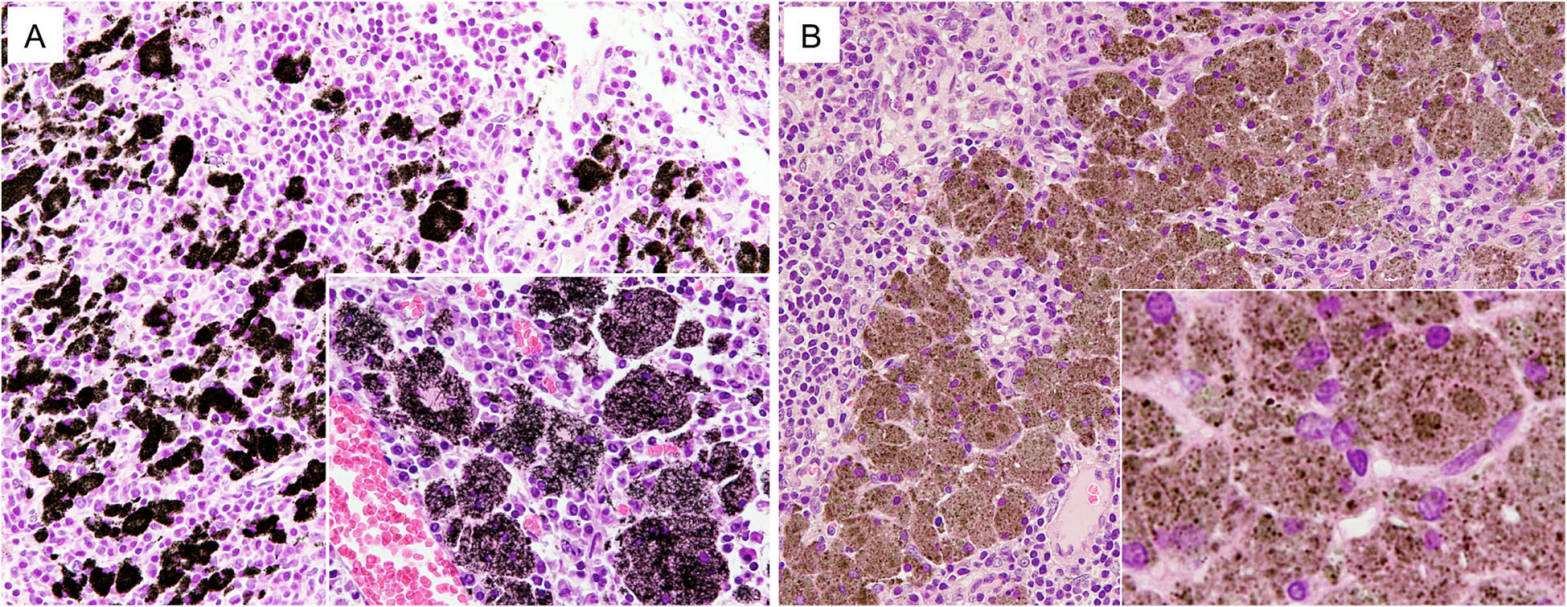
Accumulation of (**a**) MWCNT-A and (**b**) MWCNT-B in mediastinal lymph nodes

Light microscopic observation indicated that both types of MWCNTs were mostly found in macrophages in the lung alveoli and para-bronchial and mediastinal lymph nodes. In rats administered MWCNT-A the fibers were rigid and straight, while in the rats administered MWCNT-B the fibers had a coarse granular appearance filling the cytoplasm of alveolar macrophages (Fig. 2a-d). Both MWCNT-A and MWCNT-B fibers accumulated in the mediastinal lymph nodes and these fibers were similar in appearance to the fibers in the alveoli (Fig. 3a, b).

**Fig. 4:**
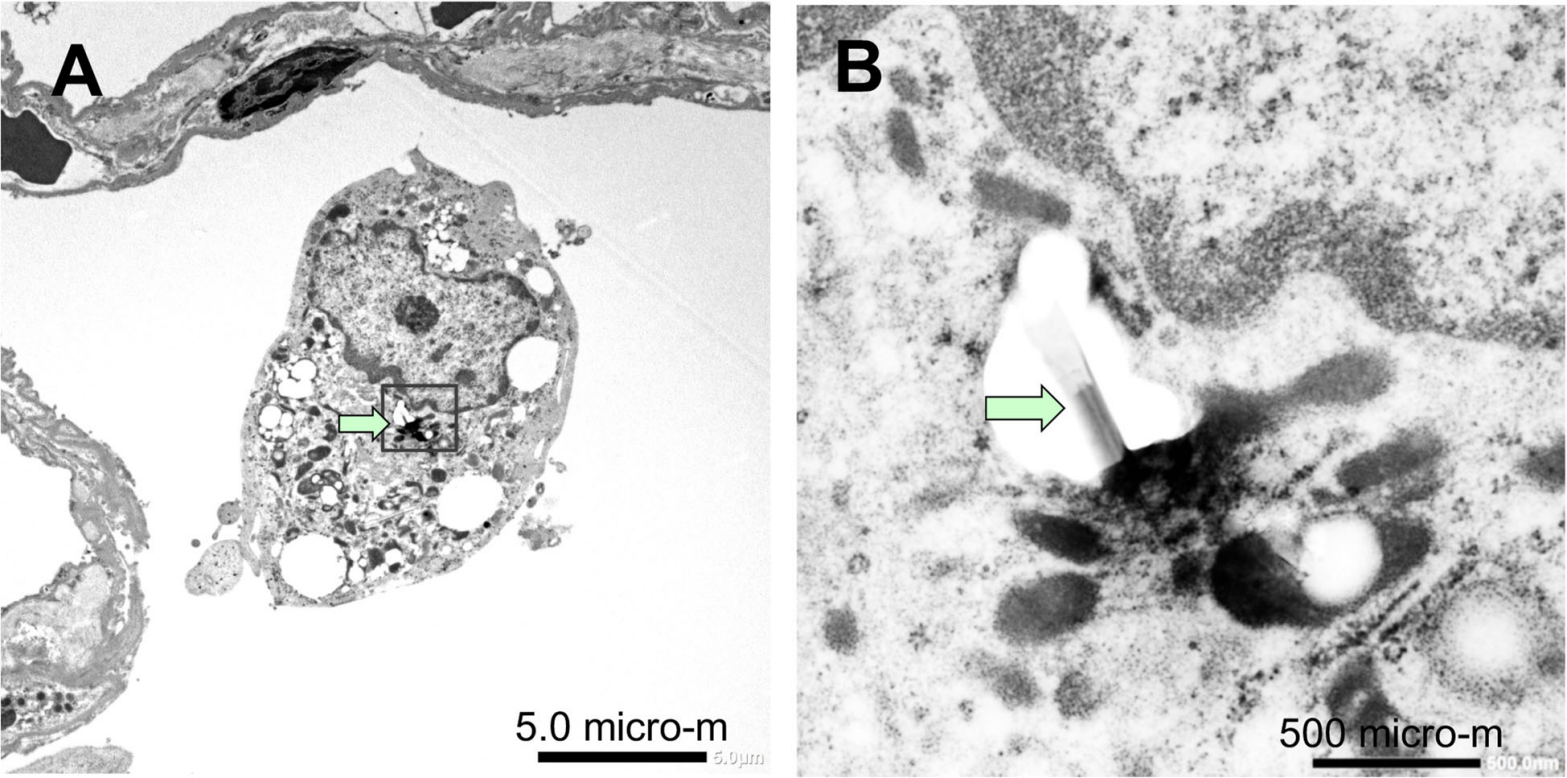
TEM image of MWCNT-A engulfed by an alveolar macrophage. **a** Fibers are phagocytosed by a macrophage in the alveolus. **b** A higher magnification of the boxed section in panel A showing a needle shaped MWCNT-A in the cytoplasm (arrow)

**Fig. 5:**
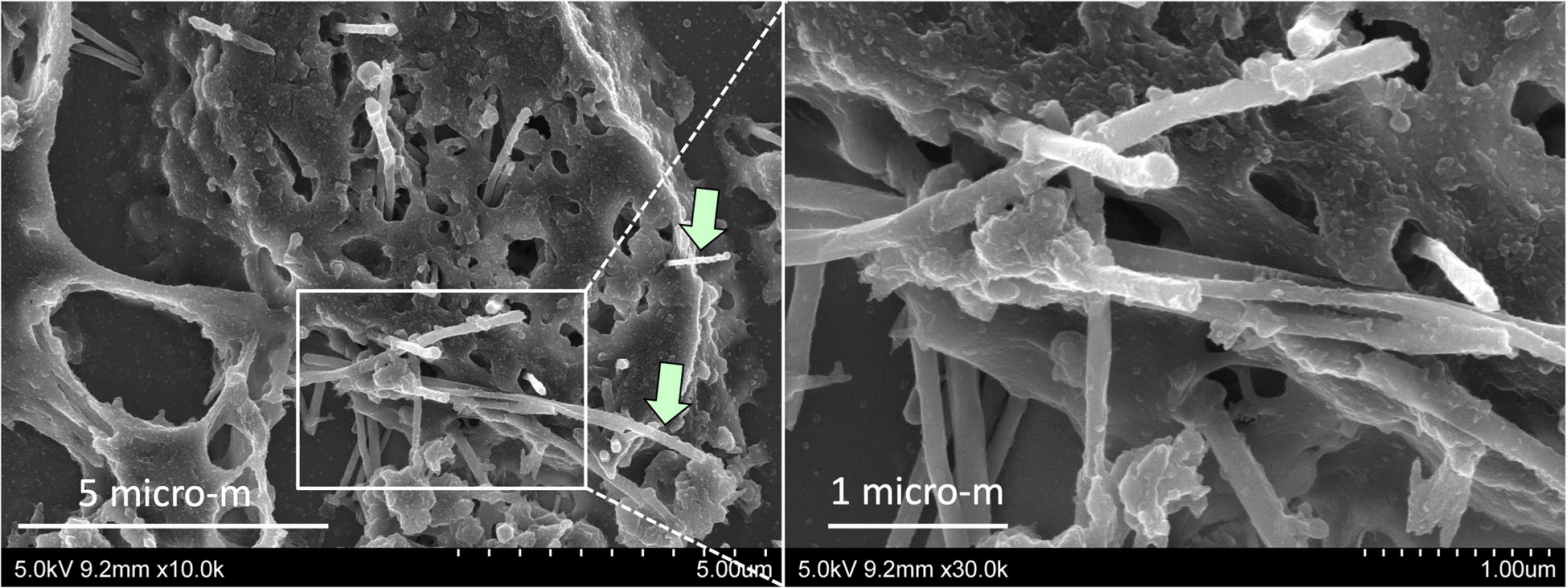
SEM image of MWCNT-A fibers engulfed by an alveolar macrophage. Arrows point to two fibers penetrating the macrophage cell membrane. The right panel shows a higher magnification of the boxed section. Straight, needle like MWCNT-A fibers are present in irregular bundles associated with the macrophage

**Fig. 6:**
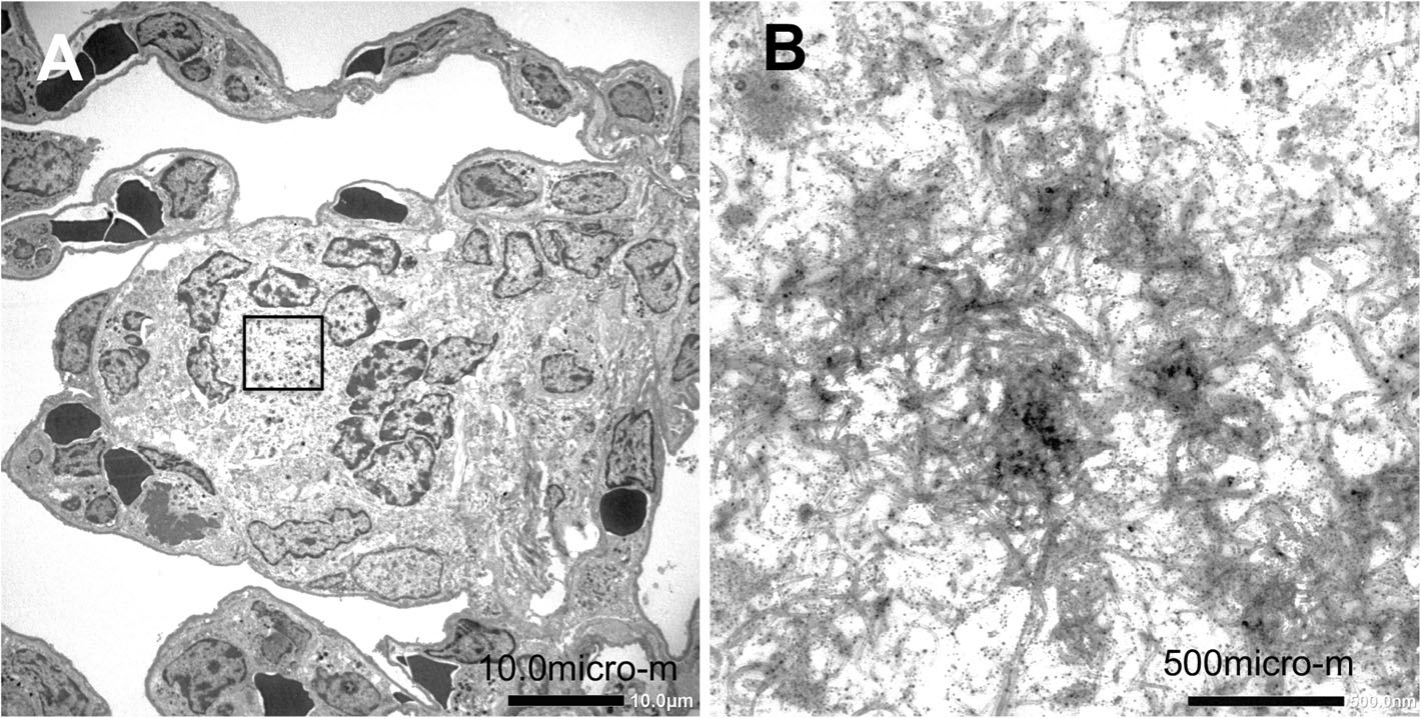
TEM image of MWCNT-B engulfed by alveolar macrophages. **a** MWCNT-B fibers are phagocytosed by macrophages forming a multinucleated giant cell surrounded by fibrotic cells in an alveolus. **b** A higher magnification of the boxed section in panel A clearly showing abundant, tangled MWCNT-B fibers

**Fig. 7:**
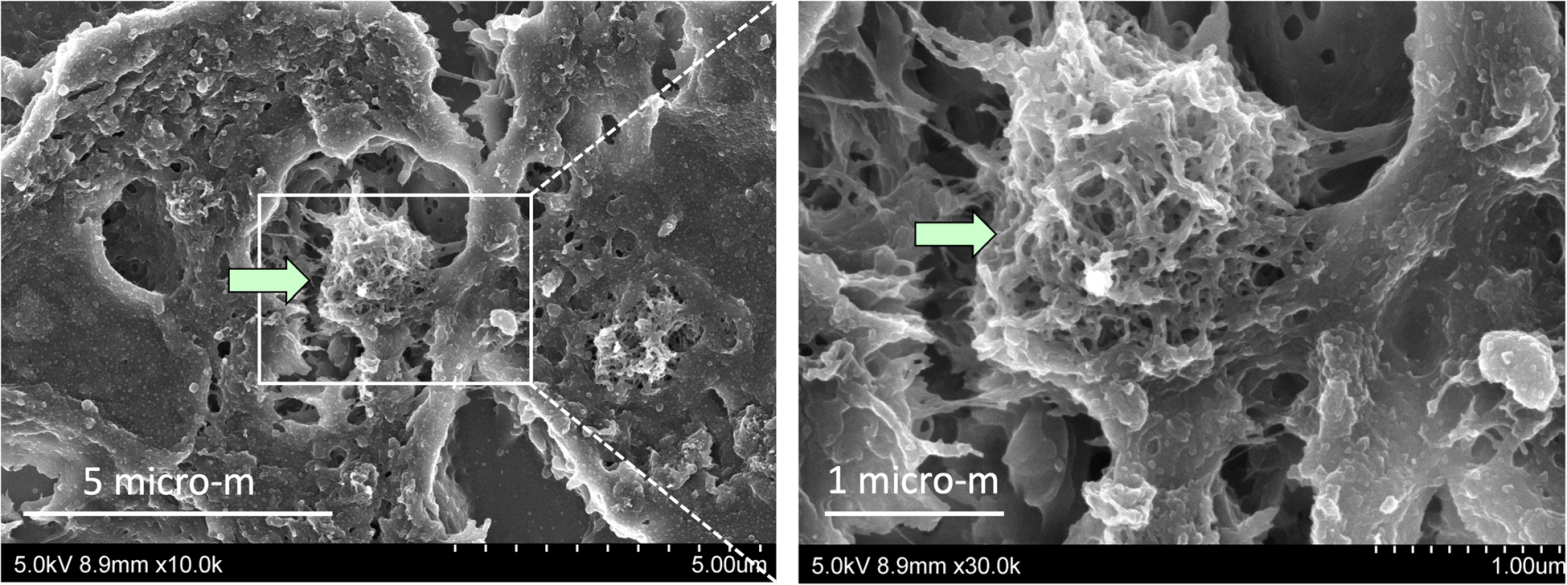
SEM image of MWCNT-B (arrow) engulfed by an alveolar macrophage. At higher magnification (right), MWCNT-B manifests as an irregularly shaped mass (arrow) in the macrophage cytoplasm

**Fig. 8:**
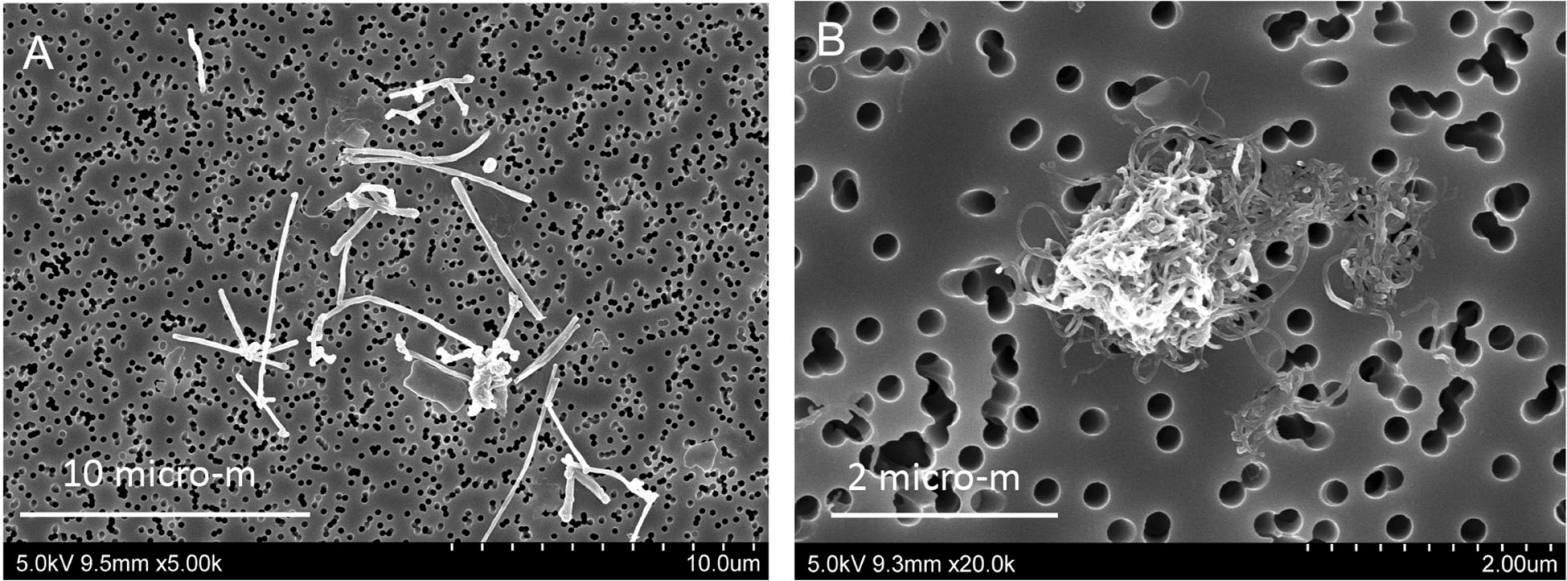
SEM images of (**a**) MWCNT-A and (**b**) MWCNT-B after dissolution of the lung tissue. **a** shows individual primary, thick straight MWCNT-A fibers. **b** shows an agglomerate comprised of thin tangled MWCNT-B fibers (see Fig. 1 in [21] for definitions of agglomerates and aggregates)

TEM and SEM observation showed rod-like MWCNT-A fibers present either singly or in irregular bundles in the cytoplasm of alveolar macrophages (Figs. 4 and 5). MWCNT-B formed irregular polygonal masses in the macrophage cytoplasm (Figs. 6 and 7). The shapes of both MWCNT-A and MWCNT-B in SEM images taken after dissolving the lung tissue were the same as in the SEM images taken of intact slide sections (Fig. 8).

### Biopersistence of MWCNTs and inflammation in the lung

Table 3 shows the amount of MWCNT-A and MWCNT-B in the lung. Both MWCNT-A and MWCNT-B were biopersistent in the rat lung with both types of MWCNTs being present primarily in granulation tissue. The fibers were present in the lungs in proportion to the amount of fibers administered, i.e., the amount of fibers in the lungs of animals administered 1.0 mg fibers was approximately twice the amount of fibers in the lungs of animals administered 0.5 mg fibers. The amount of MWCNT-B at both 52 and 104 weeks was markedly higher than MWCNT-A.

**Table 3:** Amount of MWCNTs in the lung at 52 and 104 weeks. Data is presented as micrograms MWCNT per gram of lung tissue. The lungs of 5 animals were examined for each data point

	MWCNT-A		MWCNT-B	
	0.5 mg	1.0 mg	0.5 mg	1.0 mg
52 wks	44.9 ± 6.2	94.6 ± 24.5	170.8 ± 86.3	344.3 ± 51.7
104 wks	41.6 ± 8.4	93.1 ± 17.0	135.0 ± 19.7	342.6 ± 96.9

Macrophage count was used as an indicator of inflammation (Table 4). As can be seen, macrophage count is significantly higher in the MWCNT-A and MWCNT-B administered rats compared to the vehicle control rats. There was also an increase in macrophage counts in the crocidolite administered rats compared to the vehicle control rats at 52 weeks, but not at 104 weeks. In general, there was a tendency for macrophage counts to decrease at 104 weeks compared to 52 weeks, but only in the rats administered 1.0 mg MWCNT-B was the decrease statistically significant (*p* = 0.043).

**Table 4:** Macrophage count per cm^2^ at weeks 52 and 104. The lungs of 5 animals were examined for each data point

	Vehicle	MWCNT-A		MWCNT-B		Crocidolite
		0.5 mg	1.0 mg	0.5 mg	1.0 mg	1.0 mg
52 wks	69 ± 26	258 ± 22***	268 ± 35***	412 ± 46***	499 ± 49***	130 ± 50*
104 wks	72 ± 9	235 ± 104**	367 ± 113***	393 ± 31***	426 ± 46***,#	109 ± 67

*,**,*** Different from the vehicle control at *p* < 0.05, *p* < 0.01, *p*<0.001

# Different from 52 weeks at *p* < 0.05

Compared to the crocidolite administered rats, macrophage counts were significantly higher in the MWCNT-A 0.5 mg rats at 52 weeks (*p* < 0.01) but not at 104 weeks. The macrophage counts were significantly higher in the MWCNT-A 1.0 mg rats at both week 52 and 104 (*p* < 0.01, *p* < 0.01) compared to the crocidolite administered rats. The macrophage counts were significantly higher in the MWCNT-B 0.5 mg and 1.0 mg rats at both 52 and 104 weeks (*p* < 0.001 for all comparisons) compared to the crocidolite administered rats.

Macrophage counts were higher in the MWCNT-B 0.5 mg rats compared to the MWCNT-A 0.5 mg rats at both week 52 and 104 (*p* < 0.001, *p* < 0.001) and in the MWCNT-B 1.0 mg rats compared to the MWCNT-A 1.0 mg rats at week 52 (*p* < 0.05). However, there was no significant difference in macrophage counts in the MWCNT-B 1.0 mg rats compared to the MWCNT-A 1.0 mg rats at week 104.

Overall, there was a similar trend in fiber lung burden and inflammation, both being higher in the MWCNT-B administered rats compared to the MWCNT-A administered rats. However, the fiber lung burden was approximately 3-fold higher in the MWCNT-B administered rats, while the difference in macrophage counts was much less marked, especially at 104 weeks.

### Incidence of proliferative lesions

**Fig. 9:**
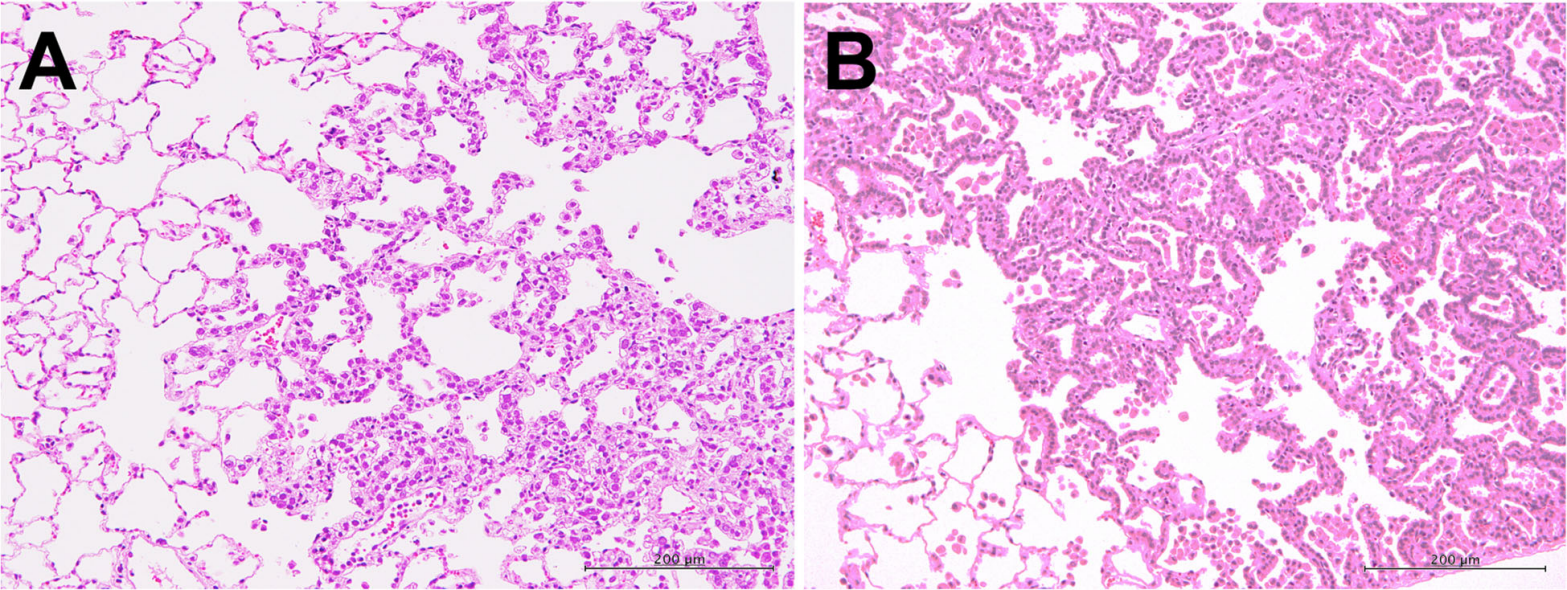
H&E section of (**a**) a lung alveolar cell hyperplasia and (**b**) a bronchiolo-alveolar cell adenoma in rats treated with 1.0 mg MWCNT-A and 1.0 mg MWCNT-B, respectively. In the region of hyperplasia in panel A, the bronchiolo-alveolar architecture is readily detectable with round to oval or cuboidal alveolar Type II cells with abundant eosinophilic cytoplasm prominently outlining the alveolar walls forming a single layer that is continuous throughout the area of hyperplasia. In the adenocarcinoma shown in panel B, the underlying alveolar architecture is obscured to various degrees, and there is a sharp demarcation of the area with high epithelial density from the surrounding tissue. For a more in-depth discussion of these lesions, see Ref [22]

**Fig. 10:**
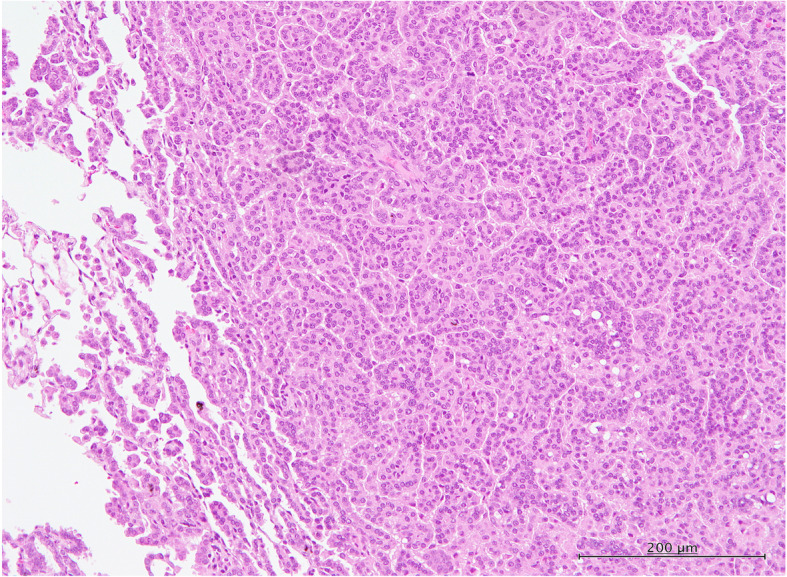
H&E section of a bronchiolo-alveolar cell carcinoma in a rat treated with 1.0 mg MWCNT-B showing invasion of the surrounding lung tissue. For an in-depth discussion of these lesions, see Ref [22]

The incidences of bronchiolo-alveolar hyperplasia (BAH), bronchiolo-alveolar adenoma (BAA), and bronchiolo-alveolar carcinoma (BAC) are shown in Table 2. In the MWCNT-B group, BAH was significantly increased in both the 0.5 mg group (6/20) and 1.0 mg group (9/20) compared to the control group (0/19), and total lung tumors, i.e., the combined incidence of BAA and BAC, in the 1.0 mg group (7/20) was also significantly higher than the vehicle control group (1/19). Neither the incidences of BAH nor total lung tumors was significantly higher in the rats administered MWCNT-A or crocidolite, however, the combined incidences of proliferative lesions, i.e., BAH + BAA + BAC in the 0.5 and 1.0 mg MWCNT-A groups (14/40) and BAH + BAA + BAC in the 1.0 mg crocidolite group (7/20), was significantly higher than the vehicle control group (1/19). The incidence of adenomas in the MWCNT-A groups (5/40 = 12.5%) and the crocidolite group (3/20 = 15%) was also higher than the historical control data of bronchiolo-alveolar adenoma incidence of the Japan Bioassay Research Center (40/699 = 5.7%), and the incidence of carcinomas in the MWCNT-A groups (4/40 = 10%) is also higher than the historical control data of bronchiolo-alveolar carcinoma incidence of the Japan Bioassay Research Center (7/699 = 1%). Figure 8a shows a typical bronchiolo-alveolar cell carcinoma in a rat treated with 1.0 mg MWCNT-A. Figure 9a shows a typical alveolar hyperplasia in a rat treated with 1.0 mg MWCNT-A; Fig. 9b shows a typical bronchiolo-alveolar cell adenoma in a rat treated with 1.0 mg MWCNT-B; and Fig. 10 shows a typical bronchiolo-alveolar cell carcinoma in a rat treated with 1.0 mg MWCNT-B.

No pleural mesotheliomas were found. Other tumors such as leukemia, pituitary tumors, mammary tumors, and scrotal malignant mesotheliomas (data not shown) were not treatment related.

## Discussion

In the present study, we used intra-tracheal, intra-pulmonary spraying (TIPS) to administer two types of MWCNTs, a straight-type MWCNT, MWCNT-A, and a flexible, tangled-type MWCNT, MWCNT-B, to the rat lung. The total fiber doses of 0.5 mg and 1.0 mg per rat were based in part on studies showing that lung burdens above approximately 1–3 mg of poorly soluble particles per gram of lung tissue can alter retention kinetics in the lung [23, 24]. Our results show that the tangled-type MWCNT, MWCNT-B, induced bronchiolo-alveolar tumors in the rat. Bronchiolo-alveolar tumors were also increased by treatment with MWCNT-A, but without statistical significance. Neither of these MWCNTs induced pleural mesotheliomas.

The primary difference in proliferative lesions in rats administered MWCNT-A and MWCNT-B was the induction of bronchiolo-alveolar cell hyperplasia: 3/20 and 2/20 in the 0.5 and 1.0 MWCNT-A groups and 6/20 and 9/20 in the 0.5 and 1.0 mg MWCNT-B groups. While hyperplastic lesions may or may not develop into tumors, cells that eventually become carcinogenic go through abnormal changes that result in development of hyperplasia, followed by development of benign tumors (adenomas), and finally development of malignant tumors (carcinomas) [25, 26]. Thus, animals exposed to carcinogens will develop all three types of proliferative lesions. Another factor that could result in increased incidence of hyperplasias and adenomas in the MWCNT-B treated rats is the higher retention of MWCNT-B in the lungs compared to MWCNT-A (discussed below). If these retained fibers have carcinogenic potential, even very weak carcinogenic potential, then the induction of the hyperplasia-adenoma-carcinoma process would be weak but continuous. The results shown in Table 2 are in accord with this possibility: the number of rats with hyperplasia, adenoma, and carcinoma in the MWCNT-B groups (0.5 and 1.0 mg combined) was 15, 9, and 3, respectively, and the number of rats with hyperplasia, adenoma, and carcinoma in the MWCNT-A groups (0.5 and 1.0 mg combined) was 5, 5, and 4, respectively. It is likely that if these rats survived for another year, the incidence of carcinoma, the last stage of cancer development, would be higher in MWCNT-B treated rats as at least some hyperplasias and adenomas continued to progress into carcinomas.

The lack of apparent carcinogenicity of MWCNT-A in this initial study does not indicate that MWCNT-A is not a carcinogen in rats. The incidence of tumors in the MWCNT-A treated group was similar to that in the crocidolite asbestos treated group. Since crocidolite asbestos is a known carcinogen in rats (and humans), this suggests that MWCNT-A could be carcinogenic in rats. Also, induction of total proliferative lesions, hyperplasias + adenomas + carcinomas, in the MWCNT-A and crocidolite groups was significantly higher than the controls: 14 of 40 rats treated with MWCNT-A and 7 of 20 rats treated with crocidolite had proliferative lesions compared to only 1 of 20 rats in the vehicle control group (Table 2). Therefore, experiments using a wider range of fiber doses and an increased number of animals are needed to define the carcinogenicity of MWCNT-A in rats.

It needs to be noted that the term multi-walled carbon nanotube (MWCNT) is generally used to refer to multi-walled carbon fibers with diameters of 100 nm or less: MWCNT-A has a diameter of 150 nm and is consequently not technically a nanomaterial. However, both MWCNT-A and MWCNT-B are the same type of material, being composed of multiple coaxially arranged graphene cylinders, and the MWCNT literature applies equally to both materials. Therefore, this report follows the classification strategy of previous reports using large diameter carbon fibers composed of multiple coaxially arranged graphene cylinders [16, 17] and uses MWCNT-A to refer to material A.

While crocidolite asbestos is a known lung and pleura carcinogen in humans and rats, it lacked overt carcinogenic capability in our study. In the studies used by IARC to classify all forms of asbestos as human carcinogens, rats were exposed to 2.2 to 50 mg/m^3^ crocidolite asbestos (see Table 3.1, 3.2 pp. 261–270 in [7]). Three of these studies exposed rats to crocidolite for 24 months: altogether 1/121 rats developed mesothelioma and 20/121 rats developed lung tumors. These results are similar to our results using TIPS administration of crocidolite asbestos.

MWCNTs can be divided into two general subtypes: tangled and straight. Studies in rats using intraperitoneal injection of straight-type MWCNTs [15–18], an intermediate-type of MWCNT [18], and tangled-type MWCNTs [14–17] showed that in the peritoneal cavity the straight-type MWCNTs had the highest carcinogenic potential, the intermediate type MWCNT had a lower carcinogenic potential than the straight-type MWCNTs, and the tangled-type MWCNTs had the lowest carcinogenic potential. Two of these studies used MWCNTs with diameters similar to the MWCNT-A used in our present study. In these studies, these larger diameter straight-type MWCNTs had less carcinogenic potential than the smaller diameter straight-type MWCNTs [16, 17]. Three other studies administered MWCNT-7 or MWCNT-N (both are straight-type MWCNTs) to rats via the airway: Administration of 1.0 mg/rat of MWCNT-N using TIPS resulted in the development of both lung tumors and mesotheliomas [12]; inhalation exposure to MWCNT-7 resulted in the development of lung tumors but not pleural mesotheliomas [8]; administration of 1.5 mg/rat of MWCNT-7 using TIPS resulted in mesothelioma but not lung tumors (the authors argue that the early death of the treated rats due to the rapid development of mesotheliomas precluded the development of lung tumors) [13]. These results are consistent with the intraperitoneal studies: straight-type MWCNTs are potential carcinogens. However, to date, no studies have tested the carcinogenic potential of tangled-type MWCNTs administered via the airway. Our results are in contrast to the intraperitoneal studies: the tangled-type MWCNT, MWCNT-B, was a more potent carcinogen in rats than the straight-type MWCNT, MWCNT-A, when the test materials were administered via the airway.

Administration of 1.5 mg of the straight-type MWCNT, MWCNT-7, to the rat lung using TIPS resulted in the development of pleural mesothelioma [13]. We have also used MWCNT-7 as a control in other studies, and administration of 0.5 and 1.0 mg MWCNT-7 via the airway also results in the development of pleural mesothelioma in rats (studies in progress). In contrast, administration of 1.0 mg of the large diameter straight-type MWCNT-A and the tangled-type MWNCT-B by TIPS to the rat lung did not cause development of pleural mesotheliomas. Since the superior mediastinal, intercostal, paraesophageal, and extrathoracic lymphatics drain the pleural space [27], the presence of MWCNT-A and MWCNT-B fibers in the mediastinal lymph nodes indicates that these fibers had passed through the pleural cavity; although, because the fibers that passed from the lungs into the pleural cavity did not accumulate in the pleural space, we were unable to detect fibers in the pleural cavity. These results suggest that tangled-type MWCNT-B and high diameter MWCNT-A have lower carcinogenic potential in the pleural cavity than straight-type MWCNT-7 when the MWCNT fibers are administered via the airway. These results are in agreement with the intraperitoneal injection studies referenced above.

The iron content of MWCNT-A and MWCNT-B is strikingly different: MWCNT-A has an iron content of approximately 0.001% while MWCNT-B has an iron content of 1.1%, a difference of more than 1000-fold. While iron content has not been found to be associated with MWCNT induction of mesothelial tumors in the peritoneal cavity [17], it is possible that iron content may have a role in MWCNT-mediated carcinogenicity in the lung [28]. Another notable difference between MWCNT-A and MWCNT-B fibers is the number of coaxial graphene cylinders that compose the fibers. With a diameter of 150 nm and 213 walls, the outer most cylinder of MWCNT-A has a surface area of approximately 1% of the total surface areas of the graphene cylinders. With a diameter of 7.4 nm and 6–7 walls, the outer most cylinder of MWCNT-B has a surface area of approximately 20% of the total surface areas of the graphene cylinders. Since these materials were not modified, the surface areas of the graphene cylinders are approximately proportional to the amount of material composing the cylinder. This means that for the same amount of material, the surface area of MWCNT-B is approximately 20-fold higher than the surface area of MWCNT-A. Since surface area is considered to be an important factor in MWCNT toxicity in the lung [29], the greater surface area of MWCNT-B may have played a role in the lung carcinogenicity of MWCNT-B.

Another critical factor is the structure of the agglomerate formed by MWCNT-B (Fig. 8). This agglomerate is similar to that formed by the MWCNTs known as Baytubes that were characterized by Jürgen Pauluhn in a 13-week subchronic study [30]. Pauluhn concluded that the void-space present in these agglomerates resulted in volumetric overload of alveolar macrophages at lower particle mass than is the case for particle masses with little void-space (such as MWCNT-A), and that this volumetric overload resulted in increased biopersistance of the Baytubes in the lungs of the exposed rats, resulting in chronic inflammation and pulmonary toxicity. In another study, mice were instilled with a variety of MWCNTs, including thin tangled MWCNTs, and sacrificed 1 year after exposure [31]. In contrast to our results, at 1 year thin tangled MWCNTS induced varying degrees of histopathological changes in the lung, but no hyperplasia was reported to have developed in the lungs of these mice. The studies cited here, however, were not two-year studies, and consequently the carcinogenicity of the MWCNTs in the experimental animals was not determined in either of these studies. Still, it is possible that pulmonary overload may have contributed to the lung carcinogenicity of MWCNT-B in our study, and that the carcinogenic effect of this thin tangled MWCNT may be rat specific. This possibility requires further investigation.

Finally, the absolute amount of MWCNT-B retained in the lung was much higher, approximately 3-fold higher, than MWCNT-A. This much larger fiber burden is very likely to be due to the type of agglomerate formed by MWCNT-B, discussed above, and is arguably the primary factor in MWCNT-B induced lung carcinogenicity. However, the difference in inflammation, as indicated by macrophage count, was much less striking than the difference in the lung fiber burden of MWCNT-A and MWCNT-B, and suggests the possibility that enclosure of MWCNT-B in granulation tissue had two contrasting effects. One effect would be to retain the fiber in the lung, enhancing fiber-mediated lung toxicity. The second effect would be to decrease interaction of MWCNT-B fibers with macrophages, thereby decreasing tissue damage and reducing fiber-mediated lung toxicity: enclosure of MWCNT fibers in granulation tissue also inhibits MWCNT-mediated carcinogenicity in the peritoneal cavity [17]. Importantly, MWCNT-B associated iron can theoretically generate oxygen radicals via the Fenton reaction in the absence of fiber interaction with macrophages. Therefore, surface chemistry may play a key role in the carcinogenic potential of MWCNT-B fibers encased in granulation tissue. Thus, fiber shape and agglomerate formation, retention in the lung, and surface area and surface chemistry all appear to be involved in the higher carcinogenic potential of MWCNT-B in the rat lung compared to MWCNT-A.

## Conclusions

In contrast to previous intraperitoneal injection studies, tangled-type MWCNT-B was a stronger carcinogen in the rat lung than straight-type MWCNT-A. In our study, MWCNT-A was not overtly carcinogenic in the rat when administered via the airway using TIPS; however, MWCNT-A had a carcinogenic potential seemingly similar to or higher than crocidolite asbestos, indicating that the carcinogenicity of MWCNT-A in the rat lung and pleural cavity remains to be determined. Our results demonstrate that MWCNT-B is carcinogenic to the rat lung when administered via the airway, identifying this fiber as a potential human health hazard, and indicating that further studies examining its mode of action in the rat and its possible carcinogenic potential in humans are appropriate.

## Materials and methods

### Preparation of MWCNTs and crocidolite suspensions

Two types of MWCNTs, thick MWCNT-A and thin MWCNT-B, were obtained from Company C. MWCNT-A and MWCNT-B: the iron content of MWCNT-A and MWCNT-B was 0.001 and 1.1%, respectively, Table 5. Crocidolite (UICC grade, needle shaped, length 24.4 ± 0.5 μm) was provided by Dr. J. Kanno. The materials were suspended at a concentration of 250 μg/ml in 20 ml saline containing 0.5% poloxamer 188 solution (P5556; Sigma-Aldrich, St. Louis, MO, USA) [poloxamer 188 is also known as Pluronic F68 [32]]. The suspensions were homogenized 3 times for 3 min at 3000 rpm using an ultrasonic homogenizer (SONIFIER model 250, Benson). The lengths and diameters of airborne fibers prior to homogenization and in vehicle after homogenization are shown in Table 5 and Additional file 1: For each data point shown in Table 5, 200 to 300 fibers or agglomerates were measured. The suspensions were sonicated for 30 min shortly before use to minimize the formation of agglomerates.

**Table 5:** MWCNTs used in this study

	Length	Diameter	Iron Content
MWCNT-A Airborne	5.46 ± 3.15 μm	163 ± 63 nm	0.001%
MWCNT-A Vehicle	6.39 ± 3.07 μm	150 ± 43 nm	
MWCNT-B Airborne	5.11 ± 3.77 μm^a^	19.1 ± 5.2	1.1%
MWCNT-B Vehicle	1.04 ± 0.71 μm^a^	7.4 ± 2.7 nm	

^a^The length of individual MWCNT-B fibers could not be measured. The lengths shown are the lengths of MWCNT-B agglomerates

### Animals and treatment

Eight-week old male F344/rats (F344/DuCrlCrlj, Charles River Laboratories Japan, Inc., Kanagawa, Japan) were housed in the center for experimental animal science of Nagoya City University Medical School, maintained on a 12:12 h light:dark cycle, and received Oriental MF Basal diet (Oriental Yeast, Tokyo, Japan) and water ad libitum. After acclimatization for 2 weeks, rats (10-weeks old) were divided into 5 groups of 25 animals each. Rats under 3% isoflurane anesthesia were administered the test materials using the TIPS method once a week over a 7 week period (8 administrations from day 1 to day 50). Rats in group 1 were administered 0.5 ml vehicle (0.5% PF68 in saline). Rats in groups 2 and 3 were administered 0.5 ml of 125 μg/ml and 250 μg/ml MWCNT-A, respectively. Rats in groups 4 and 5 were administered 0.5 ml of 125 μg/ml and 250 μg/ml MWCNT-B, respectively. Rats in group 6 were administered 0.5 ml of 250 μg/ml crocidolite asbestos. The total doses of MWCNT-A and MWCNT-B were 0.5 mg and 1.0 mg per rat, and the total dose of crocidolite was 1.0 mg per rat. Animals were sacrificed at 52 weeks (5 rats from each group) and 104 weeks (20 rats from each group) after the first administration of test materials. The study was conducted according to the Guidelines for the Care and Use of Laboratory Animals of Nagoya City University Medical School (Nagoya, Japan) and the experimental protocol was approved by the Nagoya City University Animal Care and Use Committee.

### Tissue sampling and proliferative lesion diagnosis

The rats were anesthetized by 5% isoflurane then killed by exsanguination from the inferior vena cava. The whole lung was excised and tracheal infusion with 10 ml of phosphate-buffered 4% paraformaldehyde solution was performed. The lung, chest wall sections, major organs, and mediastinal and mesenteric lymph nodes were fixed in phosphate-buffered 4% paraformaldehyde solution and processed for histological analysis. Identification of MWCNT-A in the tissue was confirmed using a microscope equipped with a polarizing lens (PLM; SX51N-31P-O: Olympus, Tokyo, Japan).

Diagnosis of hyperplasia, adenoma, and adenocarcinoma was performed by Hiroyuki Tsuda, one of the authors, who is a board certified pathologists of the Japan Society of Toxicologic Pathology (Diplomate of JSTP) and Japan Society of Pathology. Diagnosis is consistent with the INHAND project [22]: see pages 39S - 46S for a discussion of the diagnosis of hyperplasia, adenoma, and carcinoma.

### Electron microscopy

For high magnification viewing, H&E stained slides were immersed in xylene to remove the cover glass, dried, and processed for SEM (Model S^− 4700^ Field Emission SEM; Hitachi High Technologies, Tokyo, Japan). For ultrafine viewing, the area in the paraffin block corresponding to the H&E slide was cut out, deparaffinized, and embedded in epoxy resin and processed for TEM (EDAX, Tokyo, Japan).

For measurement of MWCNTs, the area in the paraffin block corresponding to the position of the MWCNT was cut out, deparaffinized by immersion in xylene for 3 days, and the deparaffinized material precipitated by centrifugation at 12,000 rpm for 10 min. Residual paraffin was removed by a 2nd incubation in xylene overnight followed by centrifugation at 12,000 rpm for 10 min. The precipitate was reacted with alkaline C99 overnight at room temperature, then centrifuged at 12,000 rpm for 10 min. The precipitated MWCNTs were washed two times with PBS containing 0.1% Tween80. The MWCNTs were then placed on a micro-grid membrane (EMS 200-Cu; Nisshin EM, Tokyo), coated with osmic acid, and viewed by SEM. The length of MWCNT-B was determined by measuring the longest diameter of the agglomerate (Fig. 6; see Fig. 1 in [21] for definitions of agglomerates and aggregates).

### Measurement of MWCNTs in the lung

Measurement of the amount of MWCNT fibers in the lung tissue was performed as described previously [12, 33].

### Statistical analysis

Statistical significance was analyzed using a 2-sided t-test with the GraphPad QuickCals t test calculator for continuous data and Fisher’s exact test for categorical data. *p*-values < 0.05 were considered to be significant.

## Supplementary information

**Additional file 1: Additional Figure 1.** (A) Length distribution of airborne MWCNT-A prior to homogenization in vehicle and after homogenization in vehicle. (B) Length distribution of airborne MWCNT-B prior to homogenization in vehicle and after homogenization in vehicle.

## Data Availability

All data generated or analysed during this study are included in this published article.

## Abbreviations

BAA: Bronchiolo-alveolar adenoma
BAC: Bronchiolo-alveolar carcinoma
BAH: Bronchiolo-alveolar hyperplasia
MWCNT: Multi-walled carbon nanotube
SEM: Scanning electron microscopy
TIPS: Intra-Tracheal Intra-Pulmonary Spraying
