# Supplementary material for: Comparative carcinogenicity study of a thick, straight-type and a thin, tangled-type multi-walled carbon nanotube administered by intra-tracheal instillation in the rat
Source: Part Fibre Toxicol. 2020 Oct 15;17:48. doi: 10.1186/s12989-020-00382-y (PMC7559486; doi:10.1186/s12989-020-00382-y)
Supplement: Supplementary file 1 — Additional file 1: Additional Figure 1. (A) Length distribution of airborne MWCNT-A prior to homogenization in vehicle and after homogenization in vehicle. (B) Length distribution of airborne MWCNT-B prior to homogenization in vehicle and after homogenization in vehicle. [file 12989_2020_382_MOESM1_ESM.pdf]

A

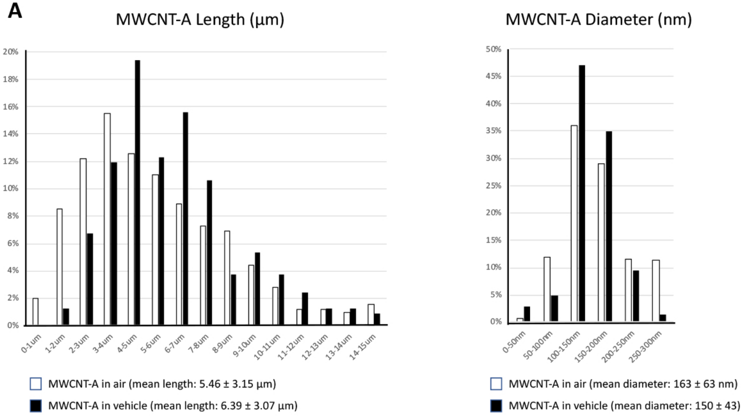

MWCNT-A Length (µm)
20%
18%
16%
14%
12%
10%
8%
6%
4%
2%
0%
0-1um
1-2um
2-3um
3-4um
4-5um
5-6um
6-7um
7-8um
8-9um
9-10um
10-11um
11-12um
12-13um
13-14um
14-15um
MWCNT-A in air (mean length: 5.46 ± 3.15 µm)
MWCNT-A in vehicle (mean length: 6.39 ± 3.07 µm)
MWCNT-A Diameter (nm)
50%
45%
40%
35%
30%
25%
20%
15%
10%
5%
0%
0-50nm
50-100nm
100-150nm
150-200nm
200-250nm
250-300nm
MWCNT-A in air (mean diameter: 163 ± 63 nm)
MWCNT-A in vehicle (mean diameter: 150 ± 43)

B

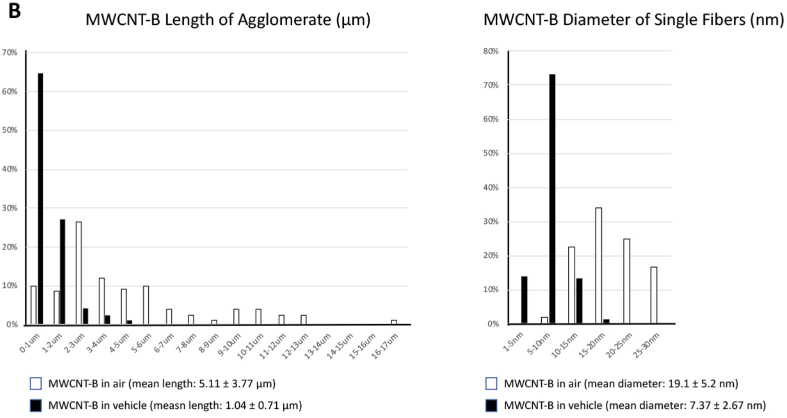

MWCNT-B Length of Agglomerate (µm)
70%
60%
50%
40%
30%
20%
10%
0%
0-1um
1-2um
2-3um
3-4um
4-5um
5-6um
6-7um
7-8um
8-9um
9-10um
10-11um
11-12um
12-13um
13-14um
14-15um
15-16um
16-17um
MWCNT-B in air (mean length: 5.11 ± 3.77 µm)
MWCNT-B in vehicle (measn length: 1.04 ± 0.71 µm)
MWCNT-B Diameter of Single Fibers (nm)
80%
70%
60%
50%
40%
30%
20%
10%
0%
1-5nm
5-10nm
10-15nm
15-20nm
20-25nm
25-30nm
MWCNT-B in air (mean diameter: 19.1 ± 5.2 nm)
MWCNT-B in vehicle (mean diameter: 7.37 ± 2.67 nm)
